# Distance to School is Associated with Sedentary Time in Children: Findings from the URBAN Study

**DOI:** 10.3389/fpubh.2014.00151

**Published:** 2014-09-23

**Authors:** Erica A. Hinckson, Les McGrath, Will Hopkins, Melody Oliver, Hannah Badland, Suzanne Mavoa, Karen Witten, Robin A. Kearns

**Affiliations:** ^1^Faculty of Health and Environmental Sciences, Auckland University of Technology, Auckland, New Zealand; ^2^Faculty of Health and Environmental Sciences, Sport Performance Research Institute New Zealand, Auckland University of Technology, Auckland, New Zealand; ^3^Faculty of Health and Environmental Sciences, Human Potential Centre, Auckland University of Technology, Auckland, New Zealand; ^4^Faculty of Medicine, Dentistry and Health Science, McCaughey VicHealth Centre for Community Wellbeing, Melbourne School of Population and Global Health, University of Melbourne, Melbourne, VIC, Australia; ^5^Social and Health Outcomes Research and Evaluation (SHORE) and Whariki Research Centre, Massey University, Auckland, New Zealand; ^6^School of Environment, The University of Auckland, Auckland, New Zealand

**Keywords:** accelerometer, commuting, neighborhood, New Zealand, physical activity, sedentary behavior

## Abstract

Sedentary behavior is associated with overweight and obesity in children, and distance to school has been negatively associated with active commuting to school. It is not known how distance to school relates to sedentary behavior in children. The aim of this study was to investigate the association between distance to school and children’s sedentary behavior during weekdays at times where children interact with the neighborhood environment. Children (5–13 years, *n* = 295) who participated in the understanding relationships between activity and neighborhoods study (2008–2010) across four New Zealand cities wore a hip-mounted accelerometer for 7 days. Minutes spent sedentary (accelerometer count <100 min^−1^) were derived for the school travel periods (0800–0859 and 1500–1559) and after school discretionary time (1600–1759). Shortest street network distance to school was calculated from residential addresses using geographical information systems and parsed into tertiles for analysis. Children completed a daily travel log including mode of transport to and from school, which was dichotomized into active (walking and cycling) and passive (motorized) modes. Children living in the second tertile of distance from school were the least sedentary during the school traveling periods (42 ± 10%, mean ± true between-child SD) compared to those living in the first or third distance tertiles (47 ± 10 and 49 ± 10%, respectively); the differences were clear and likely substantial (90% confidence limits ± 6%). Children who traveled by motorized transport were more sedentary for each of the distance tertiles (50 versus 44%, 46 versus 39%, and 54 versus 27% for first, second, and third tertiles, respectively; 90% confidence limits ± 7%). In the period of 1600–1759, girls in the third distance tertile were the most sedentary. The combined effects of 1–2 km distance from school and active commuting to school contributed to least sedentary time in children.

## Introduction

Walking and cycling to school are regarded as valuable opportunities for children to accumulate physical activity (1) and maintain healthy weight (2, 3). Aside from the physical outcomes, traveling to school by active modes provides important opportunities for children to develop confidence, navigation and local environmental awareness, risk assessment, decision making skills, and habits for lifelong participation in active travel (4). Despite these important benefits, there have been declines in the number of children actively commuting to school across many developed countries. For example, in the UK walking and cycling to school decreased from 71% in 1975/1976 to 51% in 2009, yet, the mean commute distance remained constant at 1.5 miles (5). More strikingly, US data indicate that 41% of children walked to school in 1969 and this proportion declined to 13% in 2001 (6) – despite 57% of households reporting the presence of an elementary school within 1 mile of the residence (7). Presently, the “school run” in the UK generates ~20% of automobile traffic in the mornings during term time (5), causing a substantial amount of congestion on urban road networks, localized air pollution, and traffic-related injuries (8). In New Zealand, between 1989/1990 and 1997/1998, there was a doubling in the number of school children regularly driven to school (9). This prevalence increased from 45% in 1997/1998 to 58% in 2008/2012 (10). In response to these trends, initiatives to increase active commuting to school have been implemented worldwide (11–17).

The overriding environmental factor linked to commuting to school is distance (17–22). The majority of studies report that students with shorter travel distance to school are more likely to walk and cycle compared to those who have longer travel distances (23–27). Marten and Olds (24) verified an exponential decline in children’s walking and cycling as travel distance increased; 90% of trips were walked or cycled at distances <250 m, decreasing to 10% at 3.2 km. Cumulatively, this evidence suggests that school-related active transport is associated with overall physical activity accumulation and other benefits for children, yet, this behavior has declined in recent decades.

Declines in children’s physical activity are also noted in the “critical window,” the time after school (28), where youth are more likely to choose how they spend their discretionary time. It has been observed that “technology-based sedentary behaviors” were most prevalent during these critical hours in youth aged 13–16. It was also reported that regular use of motorized transport was possibly a risk factor for reduced physical activity during those hours particularly for boys. In recent studies (29, 30), increases in sedentary behavior during the critical hours continue to be observed. Colley and associates (30) reported that boys accumulated most of their sedentary time after 15:00 on weekdays. Study findings also showed that prolonged bouts of sedentary behavior were positively associated with BMI and waist circumference whereby each additional 60 min of sedentary time was associated with a ~1 kg⋅m^−2^ higher BMI and ~3 cm higher waist circumference.

There is growing concern that youth in developed countries spend a large portion of their leisure time engaged in sedentary pursuits (31, 32). Studies in adults have shown that prolonged time engaged in sedentary behavior contributes to increased risk of all-cause mortality and cardiovascular disease mortality independent of leisure time physical activity (33, 34). While cardiovascular disease may not manifest until later in life, biological precursors for these diseases due to sedentarism may develop in youth (35). Several studies have reported that youth who were more sedentary on a daily basis had higher levels of systolic blood pressure, triglycerides, cholesterol, and/or glucose (35–38). Yet, a recent longitudinal study that investigated changes in systolic blood pressure, triglycerides, and cholesterol levels associated with television viewing in girls from 9 to 19 years old, did not report similar relationships (39). Other studies have reported significant associations between cardiovascular risk factors with TV viewing but not other types of sedentary behavior (38, 40). The pioneering work by Hamilton and colleagues (41) exemplified that sedentary behavior needs to be studied separately from physical activity and that research is needed to specifically explore factors that are associated with sedentary behavior.

Since distance to school is the overriding environmental factor associated with travel mode to school and only a few studies have examined associations between active travel to school and sedentary behavior (and these have produced unequivocal findings) (42), in this study, we explore whether school commute distance is related to sedentary behavior by sex, age, and travel modality. We consider the relationships between sedentary behavior and distance, during the journey times between 0800–0859 and 1500–1559, and after school discretionary time (1600–1759). This study is the first to investigate patterning of sedentary behaviors in relation to school travel and after school time. Results can be used to inform family based and school-based interventions to improve child health.

## Materials and Methods

Data for children aged 6–12 years were drawn from the understanding relationships between activity and neighborhoods (URBAN) study, a cross-sectional study conducted in four cities (North Shore, Waitakere, Wellington, Christchurch) in New Zealand between April 2008 and August 2010 (43). The URBAN study sought to determine associations between neighborhood walkability, physical activity, and obesity in adults and children. Details of the study design, recruitment procedures, and protocol have been published elsewhere (43). Informed consent was obtained from a parent or caregiver who participated in the URBAN study. Methods and measures specific to the current investigation are presented here.

### Neighborhood and household selection

Twelve neighborhoods were selected in each city, stratified by walkability (high/low) and ethnicity (high-Māori population/low-Māori population) at the mesh-block level (geographic census unit of ~100 households) (44). Māori are the indigenous population of New Zealand (according to the Treaty of Waitangi principles of protection, participation, and partnership researchers have a responsibility to upheld these principles in research involving Māori) (45). Walkability was calculated using geographical information systems (GIS)-derived measures of street connectivity, dwelling density, retail floor area ratio, and land use mix (ArcInfo 9.1, ESRI, Redlands, CA, USA) (46). Neighborhood selection resulted in three high walkability/high Māori, three high walkability/low Māori, three low walkability/high Māori, and three low walkability/low Māori neighborhoods within each city (Table 1). Door to door methodology was employed to recruit every *nth* household (using differential sampling rates within each neighborhood), resulting in 42 households per neighborhood. The response rate for the study was 44.8%.

**Table 1:** **Number of participants and Neighborhood NDAI^a^ Total score in the three tertiles of distance from school by neighborhood type**.

	Street network commute distance between residence and school (tertiles)		
	D1	D2	D3
	
Neighborhood type			
1	19	23	27
2	34	40	37
3	30	22	15
4	17	15	16
	
	Neighborhood NDAI total		
	
1	9	9	6
2	9	10	10
3	12	14	12
4	14	16	14

*Distance tertiles (km): D1, 0–0.8; D2, 0.8–1.9; D3, 1.9–15.6*.

*Neighborhood type: 1, low walkability–low Māori; 2, low walkability–high Māori; 3, high walkability–low Māori; 4, high walkability–high Māori*.

*^a^NDAI, neighborhood destination accessibility index score, which is a geographical information system score for measuring infrastructure support for neighborhood physical activity*.

### Participants and protocol

One child per household (if the household included a child) was asked to participate following consent from their parent or caregiver. Children were included in the study provided they were between the ages of 5 and 13 years old, were not physically disabled, and they provided assent to participate. Two subsequent visits were arranged 8 days apart. During the first visit, the study was introduced, written informed consent/assent was gained, and the accelerometer and compliance log were provided to the children. Three days later, the researcher telephoned the parents to check compliance with accelerometer wear protocols. At the second visit, the researcher collected the accelerometer and compliance log, and measured participants’ height and weight.

### Measures

#### Sedentary behavior

Time spent being sedentary was objectively measured with the Actical accelerometer (Mini-Mitter, Sunriver, OR, USA) fitted to an elastic waistband and worn above the right hip. The units have been shown to be reliable and valid in children (47, 48). Prior to distribution, the units were tested for functionality and set up to record data in 30-s epochs. Participants were instructed to wear the monitors during waking hours, but remove them when participating in water-based activities and contact sports.

Actical Export File (Version 02.10) listings for each participant were read and plotted for checking using SAS (Version 9.2; SAS Institute, Cary, NC, USA). During data collection, event markers were used to monitor delivery and collection of the devices to ensure that data included for analysis belonged to the children and not the research officers when delivering and picking up the accelerometers. To check whether the accelerometers were faulty, SAS activity printouts were checked for excessive accelerometer counts accumulated during normal sleep from 0000 to 0500 hours. Data from faulty accelerometers were removed from analysis. The threshold for sedentary time was set to <100 counts per minute (49). To maximize data inclusion and avoid bias, as in our previous work with adults (50), we opted for a 60-min non-wear time and 10-h/day wear time for inclusion in daily analyses, which typically results in inclusion of ~85% of children with at least 4 days of valid activity data (51). Non-wear time was defined as 60 min or more of consecutive 0 counts (50, 52, 53). Data collected during weekend days and school holidays were excluded. With parental assistance, children completed a compliance log for the duration of the accelerometer data collection period. Each day, participants recorded the times they rose and went to bed, times the accelerometer was removed, and activities the participant engaged in during monitor removal. Information gathered from the compliance logs assisted with confirming accelerometer data start and end times.

#### Distance to school

The shortest street network distance from each residential address to the relevant school was calculated using the OD Cost-Matrix function in ArcGIS Network Analyst, Version 9.2 (54). Calculated distances were then split into tertiles based on data distribution. The categorization of data into these distances aligned with previous research (27).

#### Travel mode

Children’s mode of transport to and from school for the 7-day monitoring period was entered in a travel log by the child, with parental assistance. Travel modes were classified as passive (car or bus) or active (walk, bicycle, skateboard, scooter). Even though physical activity is accumulated before and after bus use, we included bus transport as passive in accordance with previous research (14, 15). For every child, travel mode before and after school on each day was accounted for in the analysis (see below).

### Analyses

All analyses were performed with the statistical analysis system (Version 9.2, SAS Institute, Cary, NC, USA). Generalized linear mixed modeling, realized with Proc Glimmix, was used to estimate factors affecting proportion of time spent in sedentary behavior. The dependent variable was hourly observations of the number of each child’s sedentary minutes (i.e., minutes in which the accelerometer count was <100), expressed as a proportion of the number of minutes of recording (up to 60) for each hour of each school day the accelerometer was worn. Each hourly observation was weighted by the number of recorded minutes. The logit link function and the binomial distribution were invoked to effectively specify logistic regression, and the fact that individual sedentary minutes were not independent was taken into account by estimating an over-dispersion factor. The resulting distribution of the dependent variable is called pseudo-Bernoulli (see Example 38.1 in the Proc Glimmix documentation). The fixed effects in the mixed model included main effects to adjust to the mean levels of city (four levels), maximum daily temperature (simple numeric), and sex (two levels). Hour of the day was a main effect with levels chosen that was appropriate to the periods of the day being analyzed (0800–0859 and 1500–1559 or 1600–1759 hours). Distance from school was a nominal predictor with levels representing the tertiles of commute distance. Tertiles are a user-friendly way to present or analyze continuous variables (e.g., distance) (55). This practice also circumvents the assumption of a linear relation between the variable and the outcome of interest (55). Interactions between commute distance and travel mode (active, passive), age, sex, and individual built environment features by age were examined to provide relevant means. Random model effects were the child, the interaction of the child with before or after school periods for sedentary time and with the day of the week to account for the levels of repeated measurement on each child. The residual was specified in a manner that estimated the over-dispersion factor. Random effects and the over-dispersion factor were combined to provide between-child SD for sedentary behavior for each hour of the day.

Inferences about effects were based on probabilities of substantial magnitudes of the true values of the effects (56). Inferences were mechanistic, that is, an effect was declared “unclear” if the 90% confidence interval included substantial positive and negative values (i.e., wide confidence interval). All other effects were declared clear and are shown as the observed magnitude, with a qualitative probability of that magnitude. The descriptors used were <1%, almost certainly not; 1–5%, very unlikely; 6–25%, unlikely; 26–75%, possibly; 76–95%, likely; 96–99%, very likely; >99%, almost certainly (56). The way to interpret the findings is as follows: if the result is followed with a descriptor of “likely”, it means that there is a 76–95% chance that the true value of the statistic is practically important. The same approach can be used for the rest of the descriptors.

The threshold for substantial effects was based initially on standardization: 0.20 of the pure between-child SD derived from the child random effect in the mixed model (56). For percent of time spent in sedentary behavior, 0.20 of this SD was 2.8 (where the mean percent of each hour spent sedentary was ~50). We opted for a smallest important effect of 5% of the hour, representing ~10% of total sedentary time (57).

## Results

Two hundred and sixty eight children fulfilled the accelerometer requirements (as described above) and their data were retained for analyses (Table 2). The ethnic composition of the sample was 23% Māori/Polynesian, 12% Asian, and 65% European/Other ethnic descent. The number of participants who contributed data for analysis by hours of interest is as follows: 0800–0859, 1500–1559, 1600–1659, *n* = 222; 1700–1759, *n* = 221. Different participants contributed to the total number (*n*) per hour of interest. There were trivial differences between boys and girls in age, BMI, and distance lived from school. Travel mode data were provided by 98% of participants. The remaining 2% of data belonged to children who were absent from school due to illness but continued to wear accelerometers.

**Table 2:** **Descriptive characteristics (mean ± SD) of study participants (*n* = 268)**.

Tertiles	Female	Male
**Age (y)**		
1	6.8 ± 0.9	6.8 ± 0.9
2	9.2 ± 0.7	9.4 ± 0.6
3	11.8 ± 0.6	11.7 ± 0.7
**BMI (kg⋅m^−2^)**		
1	15.4 ± 0.8	15.1 ± 1.2
2	17.3 ± 0.8	17.3 ± 0.8
3	22.5 ± 4.3	22.0 ± 3.2
**School distance (m)**		
1	530 ± 320	590 ± 260
2	1400 ± 500	1300 ± 300
3	4200 ± 2200	4400 ± 3300

*y, years; BMI, body mass index*.

Children’s sedentary times an hour before (0800–0859) and after school (1500–1559) combined, and after school discretionary time (1600–1759) by distance from school (tertiles) are presented in Table 3. Overall, in the hours before and after school, children who lived in the second tertile of distance from school accumulated the least amount of sedentary time compared to children in the first and third tertiles. However, girls who lived closest to school spent the least amount of time being sedentary, while for boys this was true if they lived in the third distance tertile from school. Children who lived in the second tertile of distance from school accumulated the least amount of sedentary time, irrespective of travel mode.

**Table 3:** **Percentage of time children spent sedentary (mean ± SD) during school travel periods and after school periods for three tertiles of distance from school and for subgroups of sex, age, and mode of transport**.

		Street network commute distance between residence and school (tertiles)		
		D1	D2	D3
**SCHOOL TRAVEL PERIODS (0800–0859 AND 1500–1559)**				
Sex	Female	45 ± 9.7	47 ± 9.8	**52 ± 9.8**
	Male	**51 ± 12**	43 ± 11	49 ± 12
Age tertile	1	**50 ± 9.6**	41 ± 9.3	**49 ± 9.6**
	2	45 ± 12	41 ± 12	55 ± 12
	3	51 ± 9.2	**53 ± 9.2**	48 ± 9.2
Mode of transport	Active	**44 ± 9.4**	39 ± 9.1	39 ± 9.1
	Passive	49 ± 9.5	46 ± 9.5	**54 ± 9.5**
**AFTER SCHOOL (1600–1759)**				
Sex	Female	55 ± 9.8	58 ± 9.7	**65 ± 9.1**
	Male	**63 ± 12**	59 ± 13	58 ± 13
Age tertile	1	55 ± 12	56 ± 11	**58 ± 11**
	2	57 ± 10	57 ± 10	**60 ± 10**
	3	62 ± 10	63 ± 10	**65 ± 9.9**

*Distance tertiles (km): 1, 0–0.8; 2, 0.9–1.9; 3, 2.0–15.6*.

*Age tertiles (y): 1, 5.1–8.1; 2, 8.2–10.5; 3, 10.6–13.0*.

*Active: walking and cycling. Passive: motorized transport*.

*Bold: greatest proportion of sedentary time*.

During the after school discretionary time (1600–1759), the least amount of sedentary time was accumulated in children in the first and second age tertiles (Table 3). The greatest proportion of sedentary time was accumulated in the oldest children living the furthest from school. Girls living in the first tertile of distance, and boys living in the third tertile of distance were least sedentary.

Analysis of the differences in the proportion of time spent sedentary between distance tertiles by age and sex tertiles, and mode of transport are presented in Table 4. Values are presented as chances with a qualitative label, describing whether the true value of the statistic was practically important. The effects with the qualitative descriptors of “likely” and “very likely” (“almost certainly” was not observed) are used to identify practically important effects. For males and children in the first age tertile (youngest), during the period before and after school traveling time, the difference in sedentary time between the second and first tertiles of distance was likely to be important (−8.0 ± 9.4% CL and −8.3 ± 10% CL, respectively). For children in the first age tertile, the difference between third and second tertiles of distance was also important (8.2 ± 9.4% CL). The greatest difference in percent mean sedentary time, which was “very likely” to be important, was observed for children in the second age tertile for comparisons between the third and second tertile of distance. The only “likely” difference observed in the period of 1600–2000 was for girls between the third and first tertile.

**Table 4:** **Changes in percent mean sedentary time during school travel periods and after school periods for school distance by sex, age tertiles, and mode of transport^a^**.

		D2–D1		D3–D2		D3–D1	
		% Mean; ±90%CL	Magnitude	% Mean; ±90%CL	Magnitude	% Mean; ±90%CL	Magnitude
**BEFORE AND AFTER SCHOOL (0800–0859 AND 1500–1559)**							
Sex	Female	1.6; 8.3	Likely trivial	5.5; 7.9	Possibly	**7.1; 7.8**	**Likely**
	Male	−**8.0; 9.4**	**Likely**	6.3; 10	Possibly	−1.7; 10	Likely trivial
Age tertiles	1	−**8.3; 10**	**Likely**	**8.2; 10**	**Likely**	−0.1; 9.8	Unclear
	2	−4.2; 10.5	Possibly	**14; 11**	**Very likely**	**10; 11**	**Likely**
	3	2.4; 9.9	Unlikely	−4.9; 10	Possibly	−2.5; 10	Possibly
Transport	Active	−4.8; 7.6	Possibly	0.4; 11	Unclear	−4.4; 10	Possibly
	Passive	−2.9; 8.0	Possibly	**8.1; 7.0**	**Likely**	5.2; 7.6	Possibly
**AFTER SCHOOL (1600–1759)**							
Sex	Female	2.2; 6.3	Likely trivial	4.6; 5.8	Possibly	**6.8; 5.8**	**Likely**
	Male	−3.8; 10.8	Possibly	−1.4; 12	Unclear	−5.2; 12	Possibly
Age tertiles	1	0.6; 11.7	Unclear	1.8; 11	Unclear	2.5; 11	Possibly
	2	−0.1; 11.0	Unclear	3.3; 11	Possibly	3.1; 11	Possibly
	3	0.4; 11.6	Unclear	3.1; 11	Possibly	2.7; 12	Possibly

*Distance tertiles (km): 1, 0–0.8; 2, 0.9–1.9; 3, 2.0–15.6*.

*Age tertiles (y): 1, 5.1–8.1; 2, 8.2–10.5; 3, 10.6–13.0*.

*Active: walking and cycling. Passive: motorized transport*.

*Bold: greatest proportion of sedentary time*.

*Chances of the true effect: <1%, almost certainly not; 1–5%, very unlikely; 6–25%, unlikely; 26–75%, possibly; 76–95%, likely; 96–99%, very likely; >99%, almost certainly*.

*^a^Data are percent mean change in the proportion of time spent sedentary Before and after school (0800–0859 and 1500–1559) and After school (1600–1759); 90% confidence limits (±CL) between distance tertiles (D2 minus D1; D3 minus D2; D3 minus D1), and the probabilistic inference about the true magnitude of change*.

## Discussion

This is the first study to examine distance to school, and objectively measured sedentary time by travel mode in children. Overall, the combined effects of residing 1–2 km distance from school and actively commuting to school contributed to least sedentary time in children. Similarly, Faulkner and colleagues (58) observed a strong association between children’s active travel (boys in particular) residing at a distance between 1000 and 1600 m from school irrespective of neighborhood type. In this study, there was also a trend toward children residing in the second distance tertile from school being substantially less sedentary than other children, implying a non-linear relationship between school travel and sedentary behavior.

A non-linear relationship between distance to school and sedentary time indicates that other factors are mediating the overall relationship. Living further away from school may contribute toward more time getting to and from school actively leaving less time to engage in sedentary activities. There are several aspects (25) that could be mediating this relationship including specific built environment characteristics (59, 60, McGrath et al., under review), walkability around schools, and ongoing school-related active commuting initiatives (11–16).

In this study, there was a strong influence of age. Those children in the third age tertile, living in the first and second distance tertiles from school were the most sedentary. Since in 9–13-year-old children, most of the sedentary behavior occurs at home (61), a short distance to school may mean that children get home from school in a short amount of time and then spend the rest of the time engaging in sedentary activities (e.g., watching television or playing video games). During the critical hours after school, children in the third age tertile were the most sedentary. As children tend to be granted more freedom to roam and determine their leisure time activities in later years (62), they may at this age choose sedentary activities over physical activities. Gorely and colleagues (63) showed that when adolescents did not participate in active travel, they did not compensate with different physical activities, but rather filled their leisure time by engaging in technology-based and social sedentary behaviors.

Earlier research has shown that children living closer to school were more likely to engage in active travel to school (25, 27, 62, 64, 65) or access their local school playground after school (66–68). We inferred that children living closest to school (first tertile of distance) may therefore engage in less sedentary behaviors before or after school. However, our data suggested that when sedentary behavior was investigated in the hours before and after school, living a distance of less than ~0.8 km (first tertile of distance) was associated with a greater proportion of time spent sedentary before and after school (particularly for boys and younger children).

Previous studies have consistently shown that boys were more active than girls and those boys engage better with active transport (3, 58, 69, 70). In our study, even though we did not investigate distance by sex and by travel mode, we observed that girls accumulated the least sedentary time living in neighborhoods <1.9 km from school. As expected, those who lived furthest away and passively commuted were the most sedentary.

Distance to school is only one of the many factors that children and parents consider or are influenced by when making decisions about travel (27, 71) or play. There are several other contextual issues (25) that could be mediating these relationships including siblings and parental modeling. King and associates (72) identified that maternal age and parental modeling were significant correlates of sedentary behavior and not physical activity. They speculated that these correlates may have been a reflection of a decreased activity in older mothers or parental occupation with younger children, resulting in more sedentary behaviors. Other factors that may be equally important include perceptions of unsafe neighborhoods and social disorder, road traffic safety, family transport choices/options, parental attitudes, social/cultural norms, socio-demographics, walkability around schools, and lack or presence of school-related active commuting initiatives (11–16). Molnar and colleagues (73) assessed social disorder in neighborhoods using videotapes of over 15,000 block faces, and saw significant negative associations between social disorder and physical activity in youth (73). Similarly, parental anxiety about neighborhood safety has been associated with reduced physical activity in inner city children (74). Interestingly, in a national sample of mothers of preschool children, it was reported that mothers’ perception of unsafe neighborhoods was related to increased children’s TV watching but no relationship was found for perceived safety and outdoor play time (75). In a relevant review (76), road safety and “stranger danger” were identified as the main parental concerns relative to children’s safety. Consequently, parents may restrict outdoor play in the neighborhood irrespective of distance to play facilities or school. In addition, social norms of being a “responsible” parent may also play a role in the choices made regarding driving children to school (76). These choices coupled with car availability may impact on whether children actively commute to school or play facilities.

Strengths of the present study include the objective measurement of sedentary time and distance to school, and the robust sampling approach. Even though the response rate was 45%, it is higher than other similar studies with adults [e.g., 26.0% (77), 11.5% (78)] and children and adolescents [27% (79), 44% (80)]. Every effort was undertaken to ensure a good response rate (81) (e.g., face-to-face recruitment, low-participant burden, no invasive procedures, practically no risk for the children to participate in the study, a draw for a trip away was offered to parents, a report of the child’s data was provided to parents after the study and other strategies employed to improve recruitment and participation). According to Galea and colleagues (82), a low-response rate, non-participation bias or non-response bias, does not necessarily mean that there will be bias fundamental to the study. The cross-sectional nature of the study means that causal relationships cannot be established. When analyzing continuous variables, it is often useful to use tertile analysis where participants are separated into equal groups. However, threshold values for the tertiles are often not exact (equal portioning) and sometimes varying the thresholds slightly can change the results.

## Conclusion

The combined effects of 1–2 km distance from school and active commuting to school contributed to least sedentary time in children. Many children could benefit from after-school activities to reduce sedentary time during the weekdays, and those living furthest or closest from school may have the greatest need. Planners and policy makers may take this new information into account when considering location when building new schools or closing down existing schools (83–86), but caution is warranted due to the confounding factors that may have not been accounted for in this analysis. Understanding the local environment from the sedentary behavior perspective, in addition to evidence from physical activity research, is critical in the efforts to increase physical activity and increase health benefits that accumulate into adulthood. The findings can provide the basis for future sedentary behavior research.

## Author Contributions

Erica A. Hinckson developed the first draft of the manuscript. Melody Oliver and Hannah Badland contributed to the first draft of the manuscript introduction. Erica A. Hinckson, Karen Witten, Suzanne Mavoa, Hannah Badland, and Robin A. Kearns contributed to the conception and the design of the study. Erica A. Hinckson, Les McGrath, Hannah Badland, and Melody Oliver managed the data collection and database. Will Hopkins analyzed data. Erica A. Hinckson and Les McGrath contributed to data cleaning and analysis. All authors provided feedback during manuscript development. Each author has read and approved the final manuscript.

## Conflict of Interest Statement

The authors declare that the research was conducted in the absence of any commercial or financial relationships that could be construed as a potential conflict of interest.
